# Fatty acid amide hydrolase inhibition and N‐arachidonoylethanolamine modulation by isoflavonoids: A novel target for upcoming antidepressants

**DOI:** 10.1002/prp2.999

**Published:** 2022-08-26

**Authors:** Wahid Zada, Jonathan W. VanRyzin, Miguel Perez‐Pouchoulen, Samantha L. Baglot, Matthew N. Hill, Ghulam Abbas, Sarah M. Clark, Umer Rashid, Margaret M. McCarthy, Abdul Mannan

**Affiliations:** ^1^ Department of Pharmacy COMSATS University Islamabad Khyber Pakhtunkhwa Pakistan; ^2^ Department of Pharmacology University of Maryland School of Medicine Baltimore Maryland USA; ^3^ Hotchkiss Brain Institute and Mathison Center for Mental Health Research and Education, Cumming School of Medicine University of Calgary Calgary Alberta Canada; ^4^ Department of Cell Biology and Anatomy & Psychiatry University of Calgary Calgary Alberta Canada; ^5^ Department of Pharmacology, Faculty of Pharmacy Ziauddin University Karachi Pakistan; ^6^ Department of Psychiatry University of Maryland School of Medicine Baltimore Maryland USA; ^7^ Department of Chemistry COMSATS University Islamabad Khyber Pakhtunkhwa Pakistan

**Keywords:** anxiety, depression, endocannabinoids

## Abstract

Modulation of the endocannabinoid system (ECS) is a novel putative target for therapeutic intervention in depressive disorders. Altering concentrations of one of the principal endocannabinoids, N‐arachidonoylethanolamine, also known as anandamide (AEA) can affect depressive‐like behaviors through several mechanisms including anti‐inflammatory, hormonal, and neural circuit alterations. Recently, isoflavonoids, a class of plant‐derived compounds, have been of therapeutic interest given their ability to modulate the metabolism of the endogenous ligands of the ECS. To determine the therapeutic potential of isoflavonoids, we screened several candidate compounds (Genistein, Biochanin‐A, and 7‐hydroxyflavone) in silico to determine their binding properties with fatty acid amide hydrolase (FAAH), the primary degrative enzyme for AEA. We further validated the ability of these compounds to inhibit FAAH and determined their effects on depressive‐like and locomotor behaviors in the forced swim test (FST) and open field test in male and female mice. We found that while genistein was the most potent FAAH inhibitor, 7‐hydroxyflavone was most effective at reducing immobility time in the forced swim test. Finally, we measured blood corticosterone and prefrontal cortex AEA concentrations following the forced swim test and found that all tested compounds decreased corticosterone and increased AEA, demonstrating that isoflavonoids are promising therapeutic targets as FAAH inhibitors.

## Graphical abstract

In vitro screening and in vivo rodent behavioral assessments demonstrate that isoflavonoids, particularly 7‐hydroxyflavone (7‐HF), biochanin‐A, and genistein, are promising FAAH inhibitors that warrant further investigation into their use for ameliorating depressive‐like behaviors.

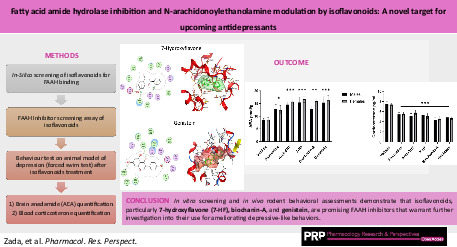

**Abbreviations**
AEA: anandamide
ECS: endocannabinoid system
FAAH: fatty acid amide hydrolase
FST: forced swim test
MOE: molecular operating environment

## 1 INTRODUCTION

Modulation of the endocannabinoid system (ECS) for therapeutic benefits has been a focus since the discovery of cannabinoid receptors and their endogenous ligands.^1^, ^2^ The two principal cannabinoid receptors, CB1R and CB2R, are G‐protein coupled receptors located in the central nervous system as well as in the periphery.^3^ The two primary endogenous ligands, anandamide (AEA) and 2‐arachidonoylglycerol (2‐AG), are produced on‐demand in the brain and activate the CB receptors.^4^ The diversity in location of the receptors, and complexity of their downstream activity in neural circuits, leads to modulation of numerous aspects of synaptic activity.^5^ Ultimately, the ECS regulates several physiological and pathological conditions including immunomodulation, pain, addictive behaviors, cognition, sociability, and stress responses.^6^, ^7^

Substantial preclinical and clinical evidence implicate ECS in depressive disorders.^3^, ^8^ CB1 receptors and the enzymes involved in AEA synthesis are highly expressed in the limbic system of the brain, including prefrontal cortex, hippocampus, amygdala, and thalamus.^9^ Moreover, the functional activity of these regions is modulated by endocannabinoids, ultimately effecting mood and emotional behavior.^10^ The ECS not only modulates neuronal circuits, but also alters several hormones that play a vital role in mood disorders^11^ and endocannabinoids can act as anti‐inflammatory agents by cyclooxygenase inhibition.^12^, ^13^ As such, modulation of the ECS has the promise to help treat and prevent depressive disorders by several mechanisms.

Several medicinal plants and their extracts have been used for generations as natural remedies to treat depression^14^ and some (Ginkgo biloba, St. John's wort, Valerian) have been validated in preclinical in vivo and in vitro studies.^15^, ^16^, ^17^ Isoflavonoids are one such example, being identified as plant‐derived compounds that target the ECS by modulating the metabolism of endocannabinoids. In particular, AEA bioavailability in the brain is controlled by the metabolic activity of the enzyme fatty acid amide hydrolase (FAAH). FAAH rapidly breaks down AEA into arachidonic acid and ethanolamide to limit AEA's ability to regulate neural transmission.^18^, ^19^ Therefore, enhancing ECS signaling can be achieved by FAAH inhibition.

We conducted this study to explore the therapeutic potential of three specific isoflavonoid compounds—7‐hydroxyflavone, biochanin‐A, and genistein—identified using an *in‐silico* drug discovery platform to detect putative FAAH inhibitors. Using a mouse model, we find that each of these compounds not only inhibits FAAH activity in vitro, but also reduces immobility time in the forced swim test, a common test for antidepressant efficacy. Furthermore, isoflavonoid‐treated animals had elevated AEA and decreased corticosterone following the forced swim test, demonstrating the efficacy of these compounds to regulate brain and behavior. Our data highlight the utility of in silico drug screening methods and identify isoflavonoids as potential novel therapeutics.

## 2 MATERIALS AND METHODS

### 2.1 In silico docking analysis

Molecular docking analysis was performed to predict the probable binding affinity between ligand and receptor and to portray distinct binding modes. The Molecular Operating Environment (MOE) software (http://www.chemcomp.com), (version 2015.10) was used to describe and predict compound binding interaction with fatty acid amide hydrolase (FAAH). The crystallographic X‐ray structure of FAAH coupled with inhibitor PF‐750 (PDB ID: 2VYA)^20^; was downloaded from the Protein Data Bank (http://www.rcsb.org). The unwanted/extra chain and water molecules were removed. Using default parameters, energy was minimized, and hydrogen was added.^21^ Genistein, biochanin‐A, and 7‐hydroxyflavone were screened through molecular docking. The molecular structures for these compounds were obtained from the PubChem database^22^ and MAPS database.^23^ The docking algorithm was authenticated by redocking the co‐crystallized ligand PF‐750 in the FAAH active site. Subsequently, a refinement induced‐fit method was performed, allowing both ligand and receptor to move freely. The positions were rescored by GBVI/WSA dG scoring function. The cognate redocking was performed to validate the docking protocol and RMSD value of co‐crystallized ligand was calculated.^24^ Ultimately, docking scores, best poses, and two‐ and three‐dimensional structures were recorded.

### 2.2 FAAH inhibitor screening assay

Analysis of FAAH inhibition was performed using the FAAH Inhibitor Screening Assay Kit (Cayman Chemicals, Cat No. #10005196) according to manufacturer instructions. This kit is a fluorescence‐based method for screening FAAH inhibitors and has been successfully used by others.^25^, ^26^ Agents were tested in triplicate and the average fluorescence of each was calculated. The percentage inhibition for each agent was calculated using the following formula:

$$\begin{array}{c} \text{Percent}\;\text{Inhibition}=\left[\left(\text{Initial}\;\text{Activity}–\text{Sample}\;\text{Activity}\right)/\text{Initial}\;\text{Activity}\right]\times 100. \end{array}$$

### 2.3 Animal studies

CD1 mice (7‐week old, 30–35 grams) were purchased from Charles River Laboratories and housed in same‐sex groups of four mice per cage. Mice were maintained on a 12:12 h natural light/dark cycle at 25 ± 2°C with ad libitum standard diet and water. Both male and female mice were used in equal proportion. All animal procedures were performed in accordance with the Animal Care and Use Committee's regulations at the University of Maryland School of Medicine.

### 2.4 Animal treatments

One week after arrival at the University of Maryland School of Medicine's animal facilities, male and female mice were assigned to one of six experimental groups for drug treatment (*n* = 8 animals per group): (i) vehicle (10% dimethyl sulfoxide in sterile water as done in Xiao et al)^27^ (ii) fluoxetine, (iii) arachidonyl serotonin (Arch‐5HT), (iv) Biochanin‐A, (v) genistein, and (vi) 7‐hydroxyflavone. Fluoxetine (10 mg/kg; Sigma‐Aldrich, Cat No. #F132) was used as a standard antidepressant comparison for all groups as done in,^28^ while arch‐5HT (5 mg/kg; Sigma‐Aldrich, Cat No. A7357) was used as a standard FAAH inhibitor as done in.^29^, ^30^ Three different doses were selected based on published studies and were tested for each of the isoflavonoid compounds, and the lowest, maximally effective dose (indicated in bold) was selected and analyzed: biochanin‐A (20 mg/kg, 30 mg/kg, 40 mg/kg; Sigma‐Aldrich, Cat No. #D2016)^31^ genistein (10 mg/kg, 20 mg/kg, 30 mg/kg based on^32^; Sigma‐Aldrich, Cat No. #G6649), 7‐hydroxyflavone (10 mg/kg, 20 mg/kg, 30 mg/kg; Sigma‐Aldrich, Cat No. #H4530).^33^ The effect was measured after chronic administration of drugs, that is, 14 days of compounds/drug treatment followed by forced swim test. The minimum, maximally effective dose based on immobility time in forced swim test was selected.

### 2.5 Animal behavior testing

Once assigned to an experimental group, mice underwent 14 consecutive days of intra‐peritoneal drug administration chronically as done in.^34^, ^35^ Mice were tested in an open field apparatus for locomotor activity 1 h before and 1 h after the administration of the last dose of drug. Mice were then tested in the forced swim test after the 14 days of drug treatment. Subsequent to the final day of treatment and testing, mice were immediately euthanized by cervical dislocation to obtain blood and brain samples for biochemical analysis.

### 2.6 Open field test

The open field test was performed as described previously^36^ with minor modifications to assess locomotor and exploratory behavior. Mice were placed into an open polycarbonate arena (50 cm long × 50 cm wide × 38 cm high) for a 60 min habituation period. Mice were then injected as described above and placed back into the arena for a 60 min testing period. After each test, the arena was cleaned with 70% ethanol solution. The testing arena received uniform low light illumination with an overhead camera to record locomotion. TopScan software (CleverSys, Inc) was used to analyze the total distance traveled over the course of the test. Data are presented as the average distance traveled in 10 min bins.

### 2.7 Forced swim test (FST)

The FST was performed as described previously.^37^ The clear polycarbonate cylinder (20 cm diameter, 30 cm high) was filled with water to a height of 15 cm and water temperature was maintained between 23 and 25°C. Mice were held by the tail and slowly lowered into the water to prevent the animal's head from submerging. Mice were video recorded by a side‐facing camera for a period of 6 min, at which point animals were removed from the water, dried, and placed back into their home cage.^38^ Time spent immobile during the last 4 min of the test was manually quantified by an experimenter blind to experimental group as described in.^37^

### 2.8 Corticosterone measurement

Blood samples were collected from mice immediately following the FST on the 14th day and centrifuged at 2000 *g* for 3 min to isolate the plasma fraction. Plasma was collected and stored at −20°C until analyzed. To determine corticosterone concentrations, the DetectX Corticosterone Enzyme Immunoassay Kit (Arbor Assays, Cat No. #K014‐H5) was used according to manufacturer instructions.

### 2.9 Anandamide quantification

Brain samples were collected from mice immediately following the FST on the 14th day and the prefrontal cortex was excised and flash frozen. Samples were stored at −80°C until analyzed. Anandamide measurement from brain samples was performed using liquid chromatography/tandem mass spectrometry as previously described.^39^ Frozen brain samples were homogenized in glass tubes containing 2 ml of acetonitrile and 5 pmol d8‐AEA. After homogenization with a glass rod, the samples were sonicated, incubated overnight at −20°C and centrifuged at 1500*g*. The proteins were removed and lipids from supernatants were transferred to a new glass tube and evaporated under nitrogen gas. The side walls of the obtained sample were washed with acetonitrile and evaporated again until completely dry. Finally, each sample was reconstituted with 200 μl of acetonitrile and stored at −80°C. Anandamide quantification was conducted by liquid chromatography/tandem mass spectrometry on a Eksigent Ekspert micro liquid chromatographer 200 coupled to an AB SciexQtrap5500 mass spectrometer, which was outfitted with a Turbo V Spray ion source at the Southern Alberta Mass Spectrometry Centre at the University of Calgary, as previously described.^39^ AEA concentration (in pmol/μl) was normalized to brain sample weight for statistical analysis and graphing.

### 2.10 Statistical analysis

Statistical analysis was performed using GraphPad Prism (version 8.0.1) software and results were considered significant if *p* < .05. In vivo and ex vivo data were analyzed by two‐way ANOVA with sex and treatment as factors. Specific group comparisons were tested using Tukey's HSD to compare the effect of treatment, as there was no effect of sex, or interaction, for any measure. Data are represented as the mean ± standard error of the mean.

### 2.11 Nomenclature of targets and ligands

Key protein targets and ligands in this article are hyperlinked to corresponding entries in http://www.guidetopharmacology.org, the common portal for data from the IUPHAR/BPS Guide to PHARMACOLOGY,^40^ and are permanently archived in the Concise Guide to PHARMACOLOGY 2019/20.^41^

## 3 RESULTS

### 3.1 In silico analysis of FAAH inhibitors

We used the molecular open environment (MOE) and performed a molecular docking analysis to determine the ability of each of the selected compounds to inhibit FAAH activity by binding to the active pocket. Within this pocket, there are four virtual binding sites: (i) a Ser241, Ser217, Lys142 catalytic triad that is responsible for the enzymatic hydrolytic activity, (ii) Gly240, Gly239, Ser241, and Ile238 within the acyl‐chain binding pocket, (iii) Phe432 and Trp531 within the membrane access channel, and (iv) Phe381 and Asp403 in the cytosolic port.

**FIGURE 1:**
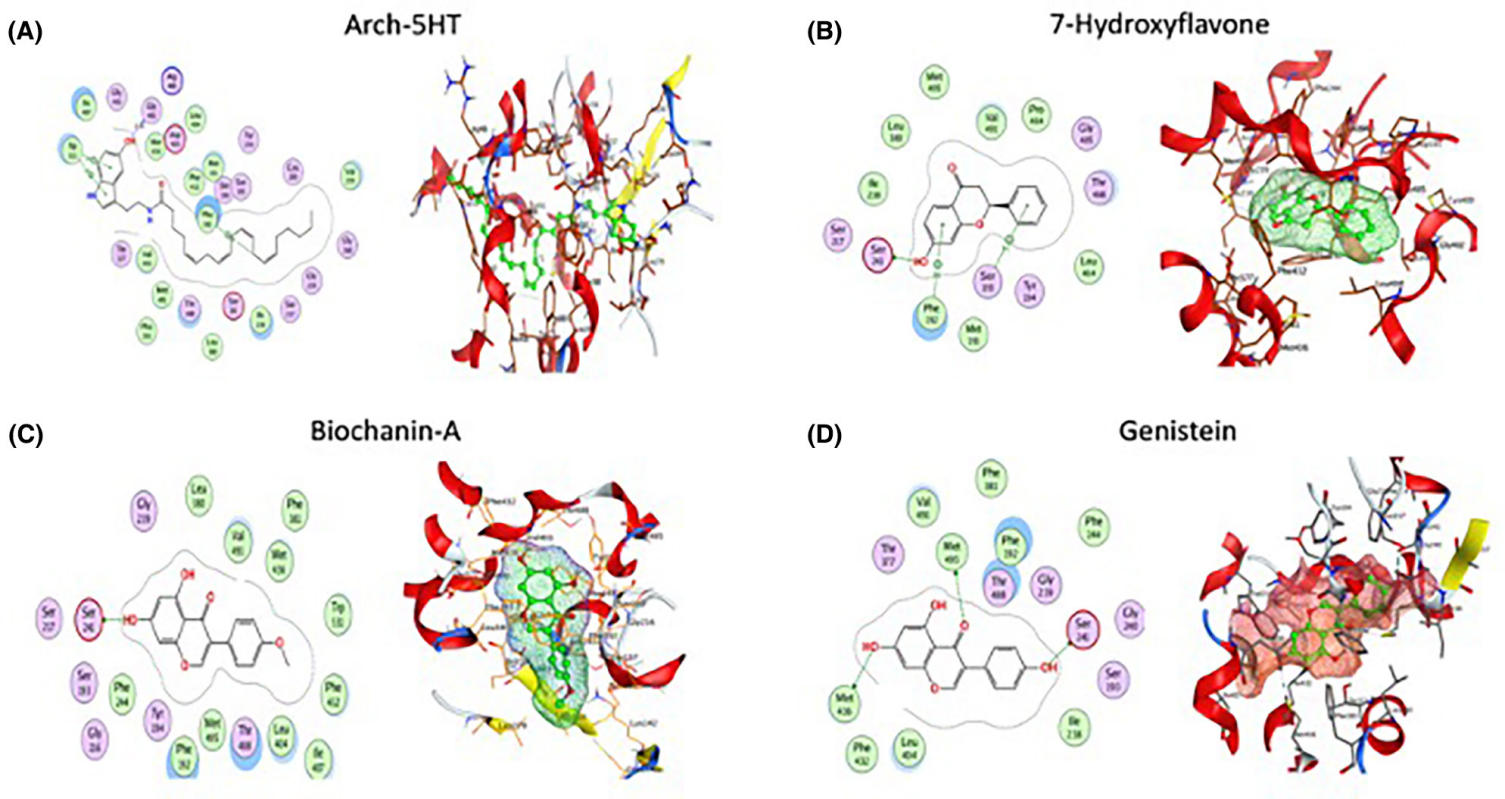
Best binding poses of isoflavonoid compounds with FAAH. Two‐ (left) and three‐dimensional (right) predicted structures of (A) arch‐5HT, (B) 7‐hydroxyflavone, (C) biochanin‐A, and (D) genistein within the active site of the FAAH enzyme. Specific hydrogen bonds between ligand and receptor are shown in green (left), while all residues involved in the enzyme/ligand interaction are shown in red/yellow (right).

7‐hydroxyflavone, biochanin‐A, genistein, and arch‐5HT were virtually docked with the FAAH crystallographic structure to study their binding properties at the active site. Each of these compounds showed comparable binding interactions to the reference ligand PF‐750. Docking simulations indicated that all three compounds had promising interactions with Ser241 within the FAAH active site (Figure 1A–D). Overall, arch‐5HT had the best docking score of −9.8445 with three Pi‐H bonds, one with Phe192 and two with Trp531, and one ‐OH bond with Gly485 with a 2.92 ^0^A. The reference ligand PF‐750 had a docking score of −8.5750. Of the three isoflavonoid compounds, biochanin‐A had the best docking score of −6.9290 followed by genistein at −6.5791 and 7‐hydroxyflavone at −6.0133.

### 3.2 In vitro analysis of FAAH inhibitory activity

**FIGURE 2:**
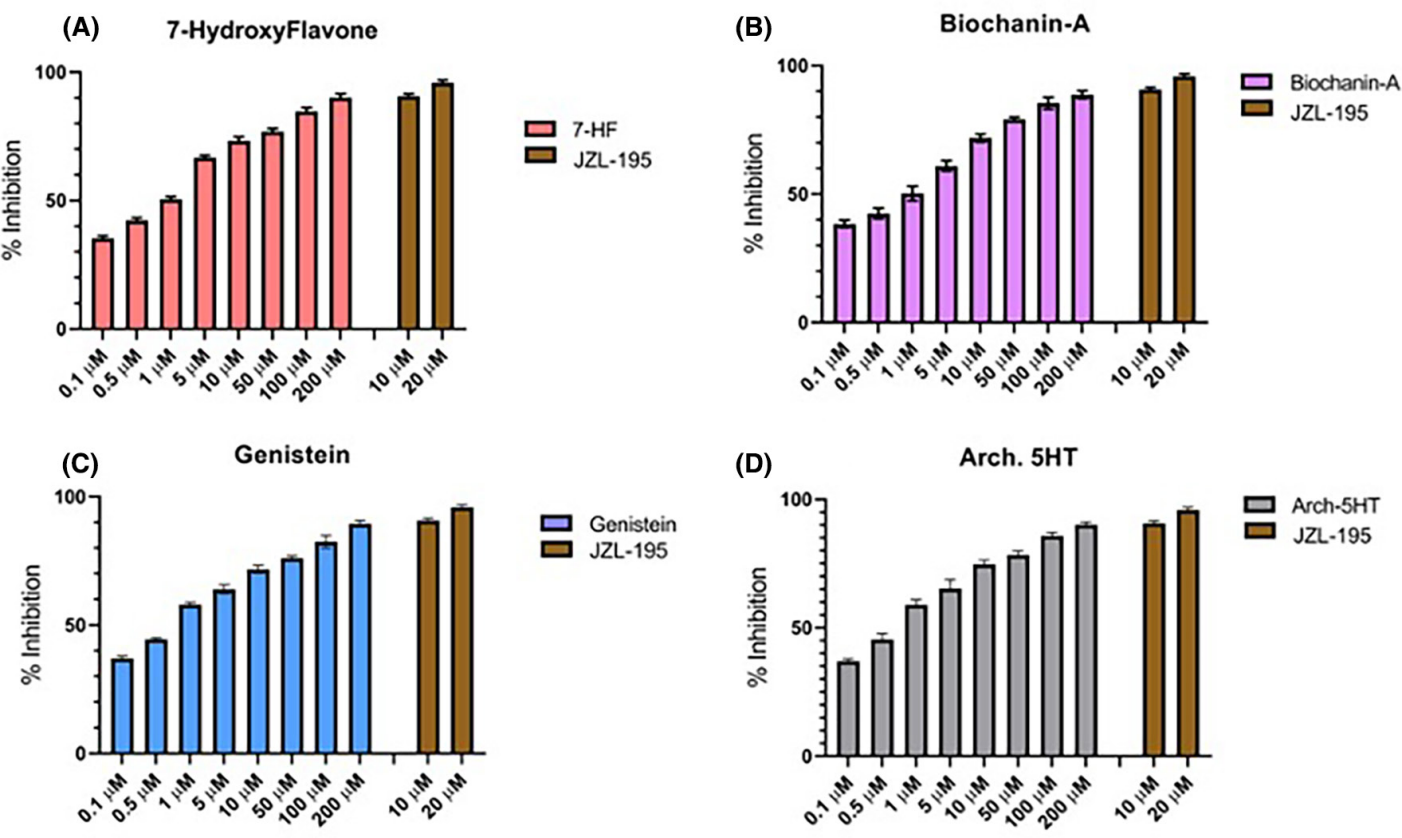
Effect of isoflavonoid compounds on FAAH activity. Percent inhibition of FAAH activity by (A) 7‐hydroxyflavone, (B) biochanin‐A, (C) genistein, and (D) arch‐5HT in a dose‐dependent manner. Data are shown as percent inhibition relative to a standard inhibitor, JZL 195.

To determine the extent to which 7‐Hydroxyflavone, Biochanin‐A, and Genistein were able to inhibit FAAH activity, we performed a FAAH inhibition assay using a commercially available ELISA‐based kit. All three compounds were tested and compared to the standard inhibitor JZL‐195 (10 and 20 μM concentration) across a dose range of 0.1 μM to 200 μM. Each showed dose‐dependent FAAH inhibition, with genistein having the lowest calculated IC50 value of 1.3 ± 0.13 μM, biochanin‐A IC50 value was 2.1 ± 0.24 μM and 7‐hydroxyflavone showing IC50 value of 2.04 ± 0.19 μM, whereas calculated IC50 value for arch‐5HT was 1.04 ± 0.13 μM. At the highest doses, all three isoflavonoid compounds were comparable in efficacy to JZL‐195 at a 10‐fold lower dose (Figure 2A–D).

### 3.3 In vivo analysis of locomotion and depressive‐like behaviors

**FIGURE 3:**
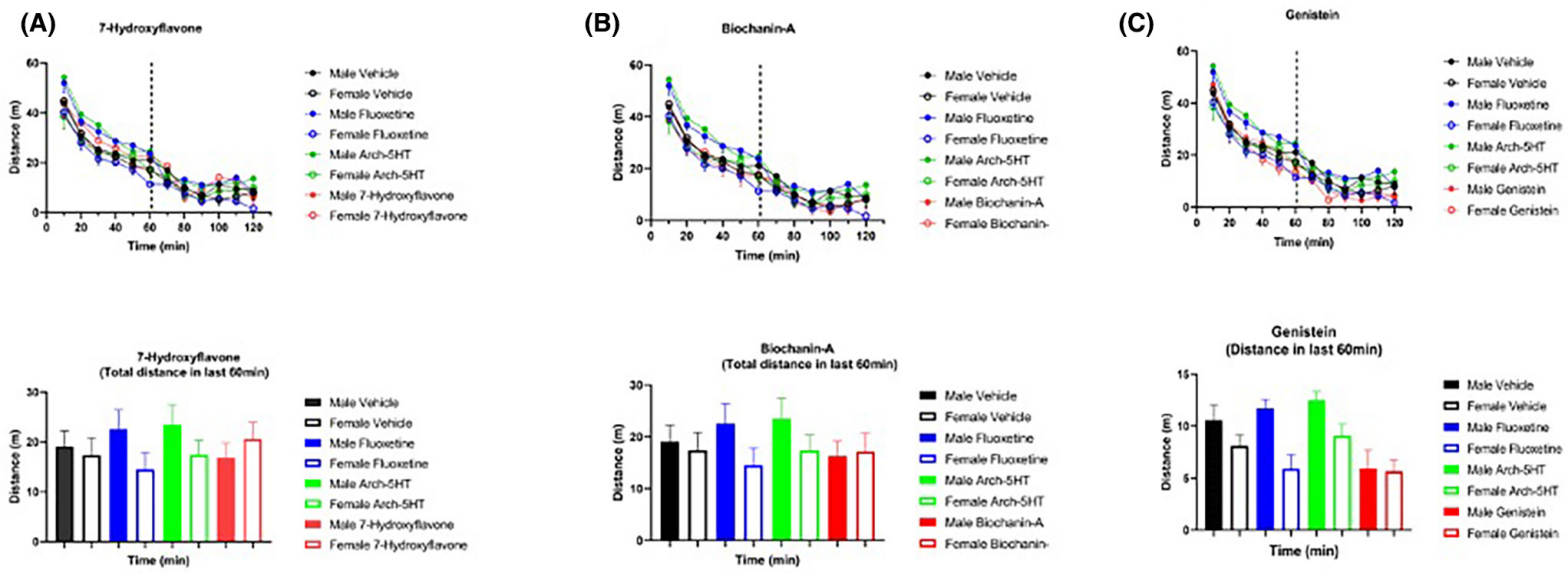
Effect of isoflavonoid compounds on locomotion. There were no treatment effects on locomotor activity in the open field test. Graphs in (A–C) are split by compound for ease of visualization; all use data from the same vehicle, fluoxetine, and arch‐5HT groups. Individual panels show the effect of (A) 7‐hydroxyflavone [20 mg/kg], (B) biochanin‐A [30 mg/kg], and (C) genistein [20 mg/kg] on distance traveled in the open field test. Data are shown as mean ± SEM. *n* = 8 per group per sex. The bar graph shows the cumulative distance covered in 60 min.

To assess the therapeutic potential of biochanin‐A, genistein, and 7‐hydroxflavone, we screened each compound in vivo using a mouse model. We used the open field test to evaluate each compound's effect on locomotion, as a proxy for psychostimulant activity.^42^ Overall, there was no significant effect of treatment (two‐way repeated measures ANOVA, main effect of treatment F [11, 84] = 1.605, *p* = .1122); 7‐hydroxyflavone‐ (Figure 3A), biochanin‐A‐ (Figure 3B), and genistein‐treated (Figure 3C) mice had similar locomotor activity when compared to either vehicle‐, fluoxetine‐, or arch‐5HT‐treated mice. Furthermore, the total locomotor activity decreased over time in all groups (two‐way repeated measures ANOVA, main effect of time F [5.058, 424.9] = 403.1, *p* < .001), indicating that none of the isoflavonoid compounds produced hyperlocomotion effects. We found no significant differences between male and female mice on locomotor activity for any of the experimental groups.

**FIGURE 4:**
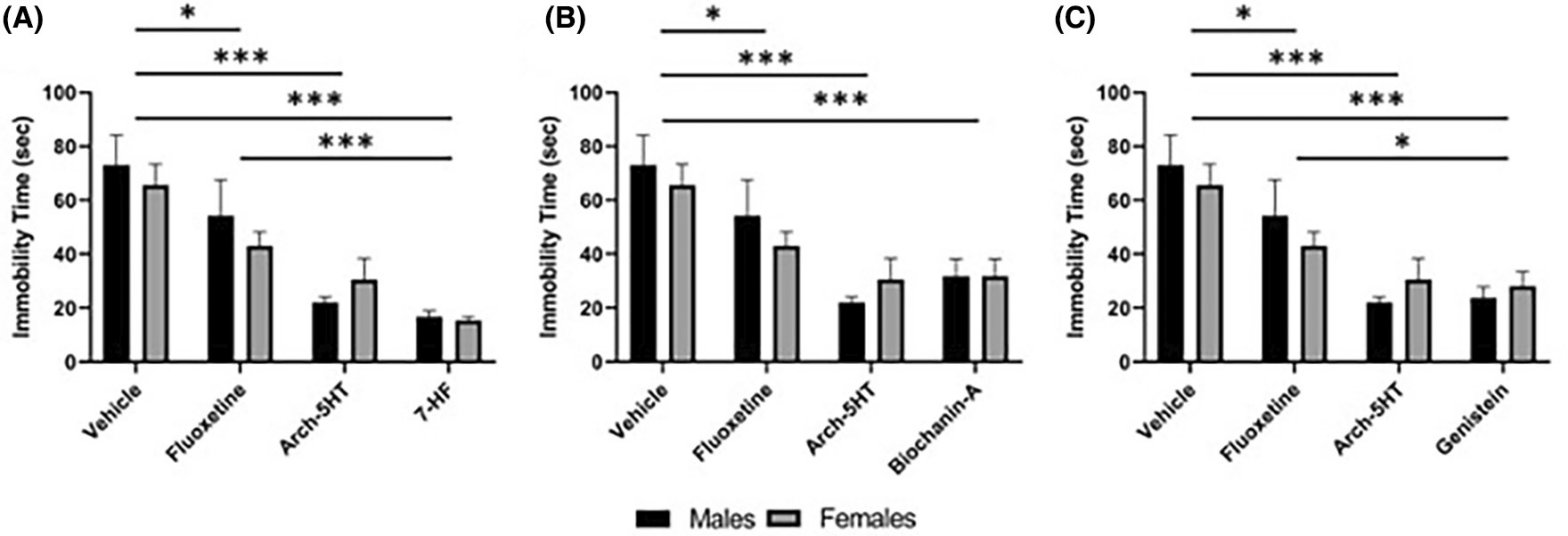
Effect of isoflavonoid compounds on immobility time in the forced swim test. All treatments significantly decreased immobility time in both male and female mice (two‐way ANOVA, main effect of treatment [F (5, 84) = 15.18, *p* < .001]). Graphs in (A–C) are split by compound for ease of visualization; all use data from the same vehicle, fluoxetine, and arch‐5HT groups. Individual panels show the effect of (A) 7‐hydroxyflavone, [20 mg/kg], (B) biochanin‐A [30 mg/kg], and (C) genistein [20 mg/kg] on immobility time in the forced swim test. Data are shown as mean ± SEM. *n* = 8 per group per sex. *p < .05, **p < .01, ***p < .001.

We then examined the effect of each isoflavonoid compound on immobility time in the forced swim test, as a measure of antidepressant efficacy. Immobility time was significantly reduced in both male and female fluoxetine (*p* = .049, Figure 4A–C) and arch‐5HT‐treated mice (*p* < .001; Figure 4A–C) compared to vehicle‐treated mice. The male 7‐hydroxyflavone group had significantly less immobility time as compared to the vehicle‐treated group (***p ˂ .001) and male fluoxetine‐treated group (*p ˂ .05). The arch‐5HT‐treated group was also significantly different from the vehicle‐treated group (*p* ˂ .01). The female 7‐hydroxyflavone‐treated mice showed similar trends compared to the vehicle‐treated group (*p* ˂ .001) and fluoxetine‐treated group (*p* ˂ .05). The arch‐5HT‐treated group was also significantly different from the vehicle‐treated group (*p* ˂ .01; Figure 4A).

Biochanin‐A treatment also resulted in antidepressant‐like effects. The male biochanin‐A‐treated mice had significantly less immobility time compared to vehicle‐treated (*p* ˂ .001) and fluoxetine‐treated (*p* ˂ .05) mice. The female biochanin‐A‐treated mice exhibited a similar trend with decreased immobility compared to vehicle‐ (*p* ˂ .001) and fluoxetine‐treated mice (*p* ˂ .05) as shown in Figure 4B.

Lastly, genistein produced the same effect as the other FAAH inhibitors, that is, it decreased the immobility time in the forced swim test. The male genistein‐treated mice and arch‐5HT‐treated mice exhibited significantly less immobility time as compared to vehicle‐treated animals (*p* ˂ .01). Female mice treated with genistein or arch‐5HT displayed a similar trend with decreased immobility compared to vehicle‐treated females (*p* ˂ .001; Figure 4C).

### 3.4 Ex vivo analysis of anandamide and corticosterone concentrations

Finally, to determine the efficacy of each isoflavonoid compound in modulating endogenous AEA and corticosterone, we collected the brains and blood from male and female mice immediately after the forced swim test. We isolated the prefrontal cortex and used mass spectrometry to determine the AEA concentrations and used a commercially available ELISA kit to measure corticosterone concentrations in the serum of blood samples.

**FIGURE 5:**
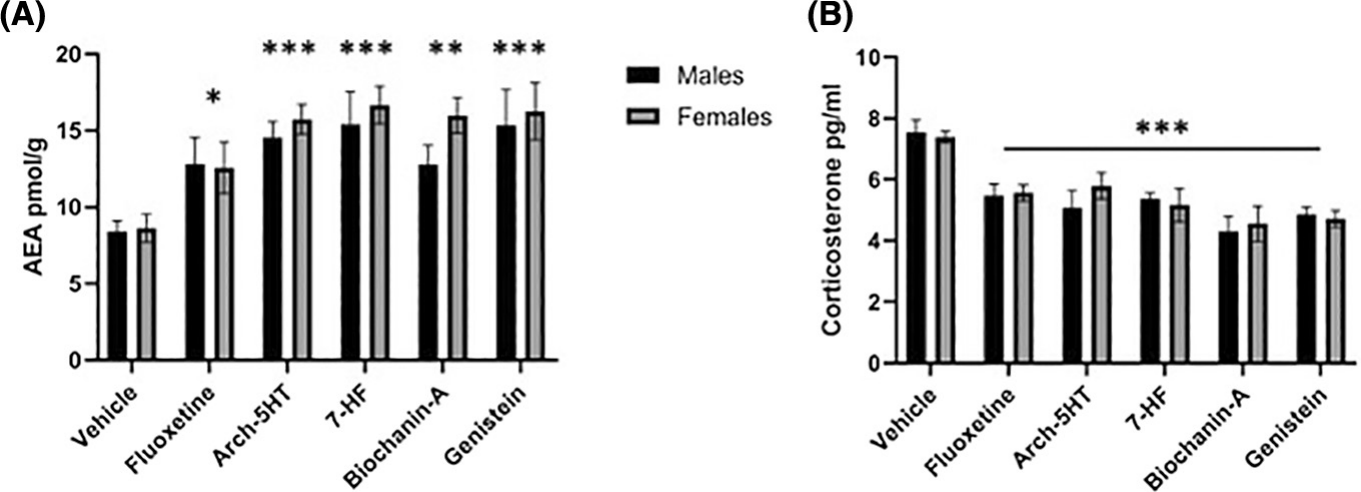
Effect of isoflavonoid compounds on prefrontal cortex AEA and blood corticosterone concentrations in an animal model of depression. (A) All treatments significantly increased AEA concentrations in the prefrontal cortex of male and female mice (two‐way ANOVA, main effect of treatment [F (5, 55) = 8.831, *p* < .001]). (B) Similarly, all treatments significantly decreased blood corticosterone concentrations in both male and female mice (two‐way ANOVA, main effect of treatment [F (5, 56) = 11.69, *p* < .001]). Data are shown as mean ± SEM. *n* = 4–8 per group per sex. *p < .05, **p < .01, ***p < .001 compared to vehicle group.

Both fluoxetine and arch‐5HT treatment increased prefrontal cortex AEA and decreased corticosterone compared to male and female vehicle‐treated mice (AEA: fluoxetine *p* < .03, arch‐5HT *p* < .001; Corticosterone: fluoxetine *p* < .001, arch‐5HT *p* < .001; Figure 5A,B). Furthermore, each of the three isoflavonoid compounds had similar effects in both sexes, significantly increasing prefrontal cortex AEA (7‐hydroxyflavone *p* < .001, biochanin‐A *p* < .003, genistein *p* < .001; Figure 5A,B) and decreasing corticosterone concentration (7‐hydroxyflavone *p* < .001, biochanin‐A *p* < .001, genistein *p* < .001; Figure 5A,B) compared to vehicle‐treated mice. We found no difference between isoflavonoid‐ and fluoxetine‐treated groups, and no difference between males and females for any of the treatment groups on either measure.

## 4 DISCUSSION

The current results demonstrate that selected isoflavonoid compounds—7‐hydroxyflavone, biochanin‐A, and genistein—have predicted interactions with the FAAH active site and are able to successfully inhibit FAAH activity in a dose‐dependent manner. Furthermore, each of these compounds significantly reduced depressive‐like behavior in male and female mice with associated increases in brain AEA and decreases in blood corticosterone concentrations.

To advance progress in drug discovery, computer‐assisted programs are often employed given their high efficiency and accuracy of predicting novel ligand‐receptor interactions. Molecular docking is one commonly used technique because of its capability to predict small ligand interactions with the active site or target binding sites.^43^ The output of these analyses, the docking score, provides quantitative values about ligand‐receptor interaction and internal energies of ligand confirmations.^44^ Molecular docking confirmed the binding of 7‐hydroxyflavone, biochanin‐A, and genistein with the FAAH enzyme. Importantly, each of these compounds had comparable docking scores to the reference ligand PF‐750 and had predicted bonding capacity with Ser241 which is a critical amino acid within the catalytic triad (Ser241, Ser217, Lys142) that is essential for FAAH's hydrolytic activity.^45^, ^46^ 7‐hydroxyflavone interacted with Ser241 suggesting a strong covalent bond. Additionally, 7‐hydroxyflavone was predicted to induce confirmational changes in the FAAH active site by bonding to Phe192 and Phe193 via Pi hydrogen bonding, potentially further inhibiting the activity of FAAH by destabilizing the optimal confirmation required for hydrolase activity. Biochanin‐A interacted with Ser241 via covalent bonding, making a stable ligand‐receptor complex, similar to previous reports.^47^ Likewise, genistein covalently bonded with Ser241 and also with Met436 and Met495 which is predicted to block the conformational changes necessary for FAAH activity upon ligand binding. Thus, all three compounds were predicted to be efficacious FAAH inhibitors.

Our data confirm the *in silico* predictions and demonstrate that all three compounds are able to inhibit FAAH activity in vitro in a dose‐dependent manner. We found that genistein was the most potent inhibitor, followed by biochanin‐A and 7‐hydroxyflavone. The data for genistein are in agreement with another study showing that genistein is a competitive FAAH inhibitor in vitro,^48^ and in vivo experiments showing FAAH inhibition by isoflavonoids in models of neuropathic pain (^49^; L^50^). However, the current study is novel in demonstrating in‐vitro FAAH inhibitory activity by genistein, 7‐hydroxyflavone, and biochanin‐A and further for determining effects on in‐vivo anandamide concentration.

Treatment with any of the three isoflavonoid compounds reduced immobility time in the forced swim test, a behavioral proxy for antidepressant efficacy. Both 7‐hydroxyflavone‐ and genistein‐treated mice had significantly lower immobility time compared to our antidepressant control treatment with fluoxetine. Importantly, none of the compounds tested had any acute effects on locomotor activity similar to previous reports,^51^ suggesting that the decrease in immobility time is not attributable to psychostimulant effects. A similar study reported that chronic treatment with genistein for 3 weeks resulted in antidepressant‐like effects in a dose‐dependent manner in two different mouse models of depression, though the hypothesized mechanism was by elevating the levels of brain monoamines and suppressing monoamine oxidase enzyme activity^52^ rather than by modulating the ECS. Additionally, an analog of 7‐hydroxyflavone reduces immobility time in the forced swim test and tail suspension test^53^ but in this instance, the marked antidepressant effect was attributed to promoting neurogenesis by tropomysin‐receptor kinase B agonist activity.^54^ The isoflavonoids 7‐hydroxyflavone and biochanin‐A have not previously been tested for antidepressant‐like activity in the forced swim test. Biochanin‐A decreases oxidative stress in a rat model of Parkinson's disease and protects dopaminergic neurons^55^ which may contribute to its anti‐depressant like effects as well.

Sustained treatment (14 consecutive days) with each of the isoflavonoid compounds produced significant increases in prefrontal cortex AEA concentrations. These findings confirm our *in‐silico* predictions and in vitro testing and demonstrate that 7‐hydroxyflavone, biochanin‐A, and genistein can each effectively inhibit FAAH in vivo. The elevation in AEA was comparable to the increase produced by arch‐5HT (an endogenous FAAH inhibitor) treatment, which has been shown to selectively inhibit FAAH in vivo to increase AEA.^56^ Consistent with our findings on fluoxetine, earlier work has indicated that fluoxetine can elevate AEA signaling in vivo,^57^, ^58^ possibly via indirect inhibition of FAAH, and that this fluoxetine‐induced elevation in AEA signaling can influence affective behaviors. The novel finding of the current study is the ability of isoflavonoids to increase anandamide in prefrontal cortex of mice.

The ECS is involved in regulating the HPA (hypothalamus, pituitary, and adrenal gland) axis and is essential to the stress response.^59^, ^60^ It is well established that depressive and anxiety disorders are characterized by HPA axis dysregulation and elevated corticosterone.^61^ More importantly, previous work has indicated that prefrontal AEA can regulate behavioral and neuroendocrine responses in the forced swim test and that declines in prefrontal AEA signaling can promote depressive‐like behaviors while elevations in prefrontal AEA signaling can produce antidepressant‐like responses and dampen stress‐induced activation of the HPA axis.^62^, ^63^ As such, our current findings mirror this established relationship between prefrontal AEA signaling and stress reactivity and further highlight the link between the ECS and the biochemical/behavioral response to stressors, while also demonstrating the efficacy of isoflavonoids to regulate these processes. Similarly, isoflavonoid treatment significantly reduced blood corticosterone concentrations in all groups. While an earlier study reported that fluoxetine treatment did not alter corticosterone concentrations following the forced swim test,^64^ we did find a decrease in corticosterone in fluoxetine‐treated animals in this study. Furthermore, the decrease observed in isoflavonoid‐treated animals is corroborated by previous studies suggesting that isoflavonoid‐modulation of FAAH activity facilitates fear extinction learning^65^ and decreases circulating corticosterone^66^ Similarly, arch‐5HT was reported to be involved in normalization of HPA axis and regulation of plasma corticosterone levels.^67^

It is important to note that we did not detect sex differences in any of the measures reported here. While depressive disorders are far more prevalent in women than in men,^68^ we found that the effects of isoflavonoid treatment on behavior, AEA and corticosterone concentrations were equally effective in non‐stressed male and female mice. While no quantitative differences in immobility time between male and female mice have been previously reported, it is possible that there may be qualitative differences in the expression of the behavior, such as head swinging behavior, that we did not quantify here.^69^

Together our data highlight the utility of using in silico techniques to identify novel protein‐ligand interactions by screening natural compounds for putative therapeutic utility. Our in vitro screening and in vivo rodent behavioral assessments demonstrate that isoflavonoids, particularly 7‐hydroxyflavone, biochanin‐A, and genistein, are promising FAAH inhibitors that warrant further investigation into their use for ameliorating depressive‐like behaviors. These data further support the involvement of the ECS in modulating the stress response and depression and will hopefully open doors to investigating novel pharmacotherapies for treatment.

## AUTHOR CONTRIBUTIONS

Participated in research design: W. Zada, J.W. VanRyzin, M.N. Hill, G. Abbas, S.M. Clark, U. Rashid, M.M. McCarthy, A. Mannan. Conducted experiments: W. Zada, J.W. VanRyzin, M. Perez‐Pouchoulen, S.L. Baglot, S.M. Clark. Performed data analysis: W. Zada. Wrote or contributed to the writing of the manuscript: W. Zada, J.W. VanRyzin, S.L. Baglot, M.N. Hill, S.M. Clark, M.M. McCarthy.

## FUNDING INFORMATION

This research was supported in part by the National Institute on Drug Abuse [R01DA039062 to MMM]; the Higher Education Commission of Pakistan [International Research Support Initiative Program to WZ].

## DISCLOSURE

The authors declare no conflict of interest.

## ETHICAL STATEMENT

All experiments were performed in accordance with the Institutional Animal Care and Use Committee’s (IACUC) regulations at University of Maryland School of Medicine in compliance with the guidelines of National Institute of Health, USA.

## Data Availability

The authors confirm that the data that support the findings of this study are available within the article andits supplementary materials, at https://doi.org/10.1002/prp2.999
